# Acupuncture for Patients With Major Depressive Disorder: An Evidence Map of Randomized Controlled Trials, Systematic Reviews, and Clinical Guidelines

**DOI:** 10.1002/brb3.71075

**Published:** 2025-11-30

**Authors:** Han Tang, Yi Gou, Xiao‐yi Hu, Zhen Luo, Wei‐juan Gang, Hong Zhao

**Affiliations:** ^1^ Department of Acupuncture and Moxibustion Shenzhen Hospital of Shanghai University of Traditional Chinese Medicine Shenzhen Guangdong Province China; ^2^ Shanghai University of Traditional Chinese Medicine Shanghai China; ^3^ Institute of Acupuncture and Moxibustion China Academy of Chinese Medical Sciences Beijing China

**Keywords:** acupuncture, clinical guidelines, evidence map, major depressive disorder, randomized controlled trials, systematic reviews

## Abstract

**Objective:**

Acupuncture is considered an effective complementary therapy for major depressive disorder (MDD), yet current findings remain inconsistent, and its overall quality is uncertain. Therefore, this study summarizes the existing evidence on acupuncture for MDD, providing an overview of the current research, identifying gaps and limitations in the literature, and offering guidance for future research.

**Methods:**

We systematically searched eight electronic databases (PubMed, EMBASE, CDSR, CENTRAL, CNKI, Wanfang, VIP, and SinoMed) and seven guideline repositories (Trip, AHRQ, NICE, NZGG, GIN, CMACPG, and NHMRC) from inception to November 15, 2024, for RCTs, systematic reviews, and clinical practice guidelines on acupuncture for major depressive disorder. Eligibility criteria were defined according to the PICOS framework. Two reviewers independently screened studies, extracted data, and assessed quality using the Cochrane Risk of Bias tool for randomized controlled trials (RCTs) and AMSTAR‐2 for systematic reviews (SRs). Key evidence and recommendations were synthesized and presented in tables and figures.

**Results:**

A total of 374 studies were identified, including 330 RCTs, 35 SRs, and 9 clinical guidelines. The RCTs generally involved small sample sizes (50 to 100 participants). The primary intervention was acupuncture combined with antidepressant medication (50%), while 79.39% of studies used antidepressants as the main control. Nearly all studies (97.88%) used changes in depression severity as the primary outcome, although the risk of bias was unclear in 80.3% of cases. Of the SRs, 97.14% reported positive findings favoring acupuncture's potential benefits, but 74.29% were rated as very low in methodological quality, lacking thorough bias assessments. Among the two acupuncture‐specific guidelines and seven broader guidelines, recommendations for acupuncture in managing MDD varied considerably.

**Conclusion:**

Evidence from RCTs, SRs, and clinical guidelines suggests that acupuncture may reduce depressive symptom severity and provide additional benefits for patients with comorbid anxiety, sleep disturbances, or somatic symptoms, particularly when used as an adjunctive therapy. However, these findings are mainly based on small‐scale trials with methodological limitations, and most guidelines recommend acupuncture only as a third‐line complementary option. Further large, high‐quality RCTs are needed to strengthen the evidence base and inform future guideline development.

## Graphical abstract

Acupuncture, particularly when combined with antidepressants, may reduce depressive symptom severity and improve comorbid anxiety and sleep disturbances in major depressive disorder. Most studies remain small and methodologically limited, underscoring the need for large, high‐quality multicenter trials.

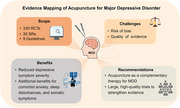

## 1 Introduction

Major depressive disorder (MDD), a common mental health condition, has emerged as a significant global public health concern (Rong et al. 2024). Approximately 280 million people worldwide are affected by MDD, with its prevalence continuing to rise (Institute of Health Metrics and Evaluation 2023). MDD is characterized by persistent low mood, loss of interest, and disturbances in appetite and sleep, which not only severely impact daily functioning but also diminish social and occupational productivity, leading to a significant socioeconomic burden (American Psychiatric Association 2013; GBD 2017 Disease and Injury Incidence and Prevalence Collaborators 2018). Current treatment strategies for MDD include pharmacotherapy and psychotherapy (Otte et al. 2016). While antidepressants have proven effective in symptom relief, they are associated with certain rates of side effects and relapse (Schmidt et al. 1986), and some patients show poor responses to conventional treatments (Dodd et al. 2021). Psychotherapy, though effective in alleviating mood disturbances, requires significant resources and extended treatment periods, which presents additional limitations (Li and Wang 2019). As a result, there is a critical need to explore complementary therapeutic approaches, particularly those with fewer side effects and the potential to address the shortcomings of pharmacological treatments.

Acupuncture, a traditional Chinese medicine (TCM) therapy, has been utilized in the treatment of MDD for many years (Kessler et al. 2001). Recent clinical studies suggest that acupuncture can effectively alleviate depression symptoms, improve mood, and enhance quality of life for patients (Zhichao et al. 2021). However, the evidence on acupuncture's efficacy remains inconsistent, with some studies reporting positive outcomes while others fail to demonstrate its effectiveness, and concerns over potential research biases have been raised (Smith et al. 2018).

Given these findings, a comprehensive evidence mapping of the existing literature on acupuncture for MDD is necessary. This paper aims to systematically review and synthesize evidence from randomized controlled trials (RCTs), systematic reviews (SRs), and clinical guidelines to identify gaps and limitations in the current body of research, providing a clear and thorough overview of the evidence surrounding acupuncture for MDD.

## 2 Material and Methods

### 2.1 Search Strategy

A comprehensive computer search was conducted across eight electronic databases: PubMed, EMBASE, Cochrane Database of Systematic Reviews (CDSR), Cochrane Central Register of Controlled Trials (CENTRAL), China National Knowledge Infrastructure (CNKI), Wanfang Data, VIP Information Service Platform, and SinoMed. The search aimed to identify RCTs and SRs on acupuncture for treating MDD from the inception of these databases up to November 15, 2024. The search was restricted to articles published in Chinese or English. For each eligible study, all available data were extracted as reported in the publication. Gray literature (e.g., conference abstracts, dissertations, or unpublished reports) was not included. Keywords used included terms related to acupuncture (e.g., acupuncture, electroacupuncture, and moxibustion) and MDD (e.g., major depressive disorder and depression). As an example, the detailed search strategy used for PubMed is presented in Table 1, while complete strategies for all databases are provided in Supporting Information.

**TABLE 1:** Search strategy of PubMed.

1	Acupuncture or Acupuncture Therapy or Auriculotherapy or Moxibustion or dry needling[MeSH Terms]
2	acupunct*[Text Word] OR acupoint*[Text Word] OR electroacupunct*[Text Word] OR electro‐acupunct*[Text Word] OR auriculotherap*[Text Word] OR auriculoacupunct*[Text Word] OR moxibust*[Text Word] OR scalpacupunct*[Text Word]
3	#1 OR #2
4	depress*[Title]
5	Depression OR Depression Disorder OR Major Depressive Disorder, Major[MeSH Major Topic]
6	#4 OR #5
7	Randomized Controlled Trial[Publication Type]
8	controlled clinical trial[Title/Abstract] OR RCT[Title/Abstract] OR random*[Title/Abstract] OR allocat*[Title/Abstract] OR assign*[Title/Abstract] OR placebo[Title/Abstract]
9	#7 OR #8
10	animal[MeSH Terms]
11	animal[Title/Abstract] OR rat[Title/Abstract] OR mice[Title/Abstract] OR dog[Title/Abstract] OR pig[Title/Abstract] OR rabbit[Title/Abstract] OR fish[Title/Abstract]
12	#10 OR #11
13	#3 AND #6 AND #9
14	#13 NOT #12

Additionally, a search was conducted in seven guideline databases, including Trip medical database, Agency for Healthcare Research and Quality (AHRQ), National Institute for Health and Care Excellence (NICE), New Zealand Guideline Group (NZGG), Guidelines International Network (GIN), Canadian Medical Association's Clinical Practice Guidelines database (CMACPG), and National Health and Medical Research (NHMRC), using search terms like “depression” or “major depressive disorder.”

### 2.2 Eligibility Criteria

Eligibility criteria were defined according to the PICOS (patient, intervention, comparison, outcome, study design) framework. We included studies enrolling patients diagnosed with depression or major depressive disorder, without restrictions on age or disease severity. Eligible interventions comprised acupuncture or related stimulation methods (e.g., manual acupuncture, electroacupuncture, moxibustion, and auricular therapy), either alone or in combination with conventional treatments. Comparators included antidepressant medications, psychological therapy, exercise, or other conventional interventions, as well as different forms of acupuncture for head‐to‐head comparisons. Outcomes of interest were changes in depression severity, adverse events, depression improvement or remission rates, quality of life, sleep, fatigue, and medication use. Eligible study designs included RCTs, SRs, and clinical practice guidelines. Detailed inclusion and exclusion criteria are summarized in Table 2.

**TABLE 2:** Inclusion and exclusion criteria.

PICOS	Inclusion criteria	Exclusion criteria
Population	Diagnosed with depression or depressive episodes; age and severity of condition not limited.	Special populations: Exclude pregnant and postpartum women, perimenopausal women, stroke patients, and those with depression caused by pain, anxiety, cancer, insomnia, post‐surgery, etc.
Intervention	Acupuncture based on acupoint selection: traditional body acupuncture, micro‐needle system (scalp acupuncture, wrist‐ankle acupuncture, abdominal acupuncture, auricular acupuncture). Stimulation methods: acupuncture, electroacupuncture, laser acupuncture, moxibustion (e.g., moxa stick, moxa cone), and auricular pressure therapy.	Experimental group using a combination of three or more traditional Chinese medicine interventions that may affect the interpretation of the specific acupuncture treatment's effect.
Comparison	Antidepressant medications: can be combined with acupuncture or used alone, including second‐generation antidepressants for comparison. Medications include selective serotonin reuptake inhibitors (SSRIs), serotonin‐norepinephrine reuptake inhibitors (SNRIs), and selective serotonin‐norepinephrine reuptake inhibitors (SSNRIs) like amitriptyline, fluoxetine, and paroxetine. Exercise: includes water‐based exercises, controlled exercise programs, balance training, Tai Chi, yoga, and Pilates. Psychological therapy: includes cognitive‐behavioral therapy (CBT), behavioral therapy, and psychotherapy. Acupuncture combined with other treatments, consistent therapies must be used: acupuncture + antidepressants vs. antidepressants; acupuncture + exercise vs. exercise; acupuncture + psychotherapy vs. psychotherapy; acupuncture + other therapies vs. other therapies. Comparison of acupuncture techniques: body acupuncture vs. scalp acupuncture; body acupuncture vs. auricular acupuncture; electroacupuncture vs. manual acupuncture. Comparison involving three or more groups (if two of these groups meet the inclusion criteria, data from two groups can be extracted).	Control group using a combination of three or more integrated intervention therapies, which may interfere with the assessment of acupuncture's effect. Comparison between special acupuncture techniques vs. regular acupuncture techniques.
Outcome	Change in depression severity: assessed using self‐rating scales (BDI) or clinician‐rated scales (HAMD, EPDS, SDS). Total number of severe adverse events at the end of treatment. Depression improvement rate: based on HAMD or other clinician‐rated scales, reported as binary outcomes. Quality of life: generally assessed using scales such as SF‐36. Changes in sleep conditions: measured using the Pittsburgh Sleep Quality Index (PSQI). Changes in fatigue levels: measured using tools like the Fatigue Assessment Instrument (FAI) or the Fatigue Scale (FS). Changes in medication use or other interventions at the end of treatment, such as adjustments in dosage or drug types, patients seeking additional treatments, and changes in the number of appointments.	
Study	Randomized controlled trials Systematic reviews Clinical guidelines	Non‐randomized controlled trials. Duplicate publications, select the most comprehensive literature. Conference abstracts, bibliometric studies, systematic review re‐evaluations, research progress reports, qualitative reviews, etc.

### 2.3 Screening and Data Extraction

#### 2.3.1 Literature Screening

All records were imported into NoteExpress software. One reviewer (H. Tang) independently removed duplicates, and two reviewers (H. Tang and Y. Gou) then screened the remaining records based on the pre‐established inclusion and exclusion criteria. Initially, irrelevant records were excluded by reading titles and abstracts. Full‐text articles of potentially relevant studies were retrieved for further screening. Any disagreements between the two reviewers were resolved through discussion; if consensus could not be reached, a third reviewer (Z. Luo or H. Zhao) adjudicated.

#### 2.3.2 Data Extraction

Data were extracted using a custom template created in Microsoft Excel. Two independent reviewers (H. Tang and Y. Gou) performed the extraction, and data were cross‐checked for consistency. The extraction form captured key information, including publication date, language, country of origin, sample size, intervention details, control measures, outcome indicators, and study conclusions. If a study reported multiple trial groups that met inclusion criteria, these were counted separately where applicable for intervention and outcome extractions. The study selection process was conducted in accordance with the PRISMA 2020 statement and is presented in a PRISMA flowchart.

### 2.4 Quality Assessment

#### 2.4.1 RCT Quality Assessment

The quality of RCTs was assessed using the Cochrane Collaboration's Risk of Bias (RoB) tool (Higgins et al. 2011), which evaluates areas such as random sequence generation, allocation concealment, blinding, outcome assessment, attrition bias, and reporting bias. Each RCT was assigned a risk rating of low, high, or unclear. Two researchers independently performed the risk assessment, with disagreements resolved through discussion with a third reviewer.

#### 2.4.2 SR Quality Assessment

The quality of SRs was reassessed using the AMSTAR‐2 tool, which consists of 16 evaluation items. AMSTAR‐2 tool is a critical appraisal tool designed for systematic reviews that include both randomized or non‐randomized studies of healthcare interventions, assisting in the identification of high‐quality systematic reviews (Shea et al. 2017).

## 3 Results

### 3.1 Study Inclusion

**FIGURE 1:**
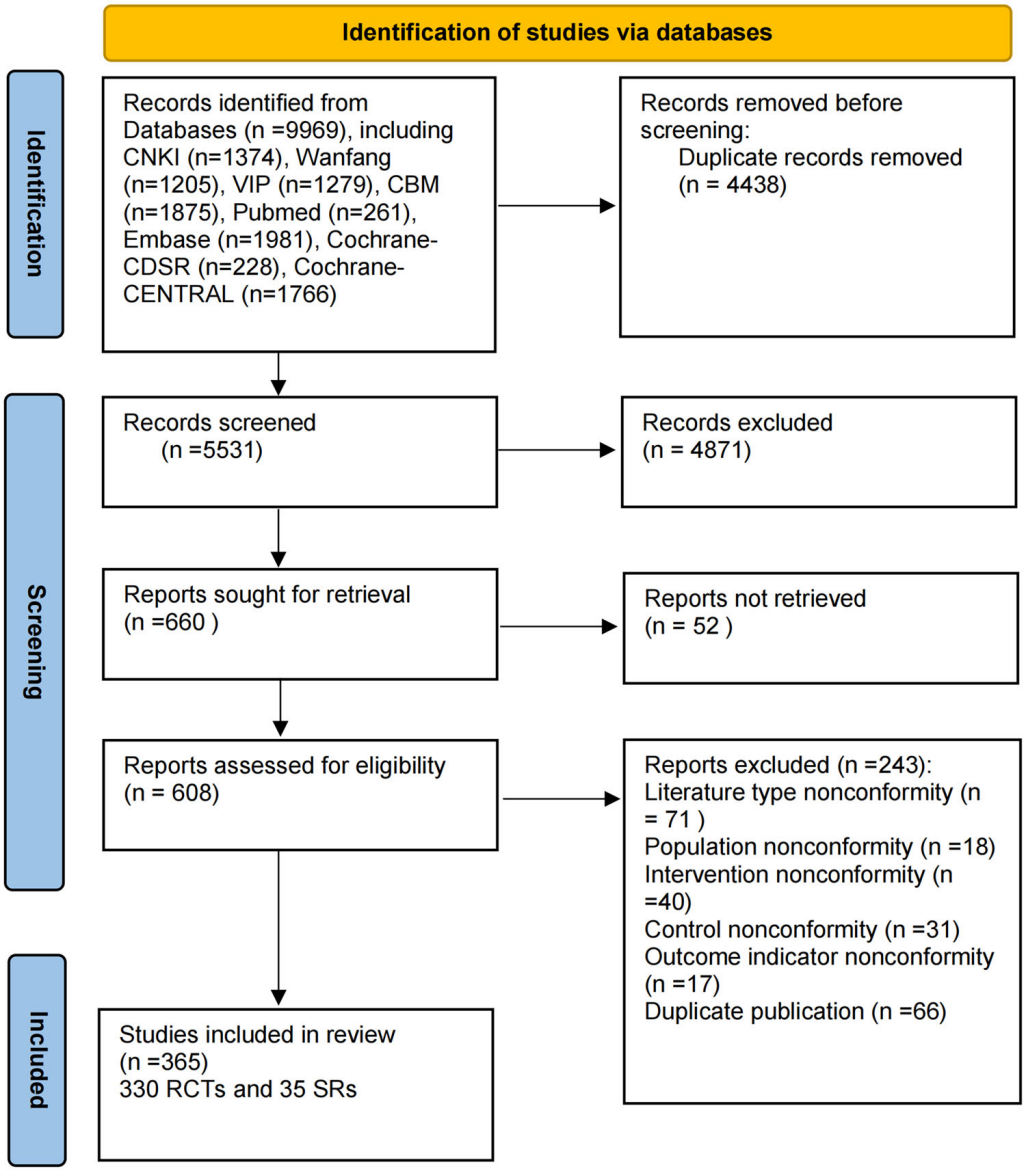
A PRISMA diagram of the literature selection.

We conducted a preliminary search across eight electronic databases, identifying 9969 potentially relevant articles. After removing duplicates, 5531 articles remained. Following an initial screening of titles and abstracts, 4871 articles were excluded for reasons such as irrelevance to the study topic. Of the 660 articles that underwent full‐text screening, 52 could not be accessed, and 243 were excluded based on our inclusion and exclusion criteria. Ultimately, 365 articles were included, consisting of 330 RCTs and 35 SRs (Yan et al. 2021; Leo and Ligot 2007; Smith et al. 2018; Armour et al. 2019; Stub et al. 2011; Nguyen et al. 2021; M. M. Xu et al. 2022; Zhou et al. 2022; Z. Zhang et al. 2016; H. Wang et al. 2008; Y. Zhang et al. 2016; Chan et al. 2015; G. Xu et al. 2022; Mukaino et al. 2005; Wang and Chi 2008; Cui 2016; Sun et al. 2008; S. Xu et al. 2022; Meng 2020; Bian et al. 2011; Wang et al. 2020; Lü 2014; L. Wang et al. 2008; Shen et al. 2014; Yuan et al. 2020; Quang et al. 2017; Xiong et al. 2009; Wang 2021; Fan 2011; Zhong et al. 2008; Peng et al. 2022; Meng et al. 2020; Tan et al. 2024; Bai et al. 2024; Li et al. 2024). The selection process is illustrated in the PRISMA flowchart (Page et al. 2021) (Figure 1).

In addition, a search of seven guideline databases initially retrieved 745 relevant guidelines, of which 693 were exported. These included 473 from Trip, 23 from AHRQ, 8 from NICE, 25 from NZGG, 41 from GIN, 25 from CMACPG, and 150 (98 of which were ultimately exported) from NHMRC. After an initial screening of titles and abstracts, further full‐text reviews resulted in the exclusion of 684 guidelines, leaving 9 for inclusion in the subsequent analysis.

### 3.2 Basic Characteristics of Included RCTs and SRs

**FIGURE 2:**
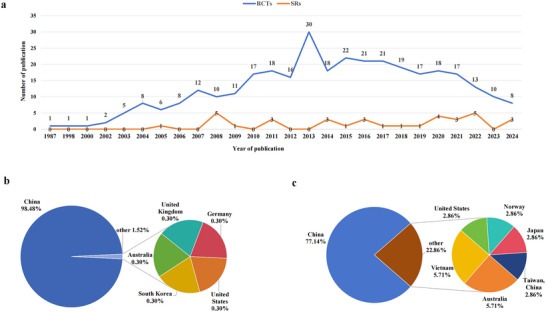
Basic characteristics of the included RCTs and SRs. (a) The number and publication years of RCTs and SRs; (b) the distribution of the first authors' countries for the RCTs; (c) the distribution of the first authors' countries for the SRs.

Three 330 RCTs on acupuncture for MDD were included, with publication dates spanning from 1987 to 2024. The number of publications increased steadily from 1987 to 2013, reaching a peak of 30 articles (9.09% of the total) in 2013. Afterward, the number of publications gradually declined, as illustrated in Figure 2a. Among these studies, 307 (93.03%) were published in Chinese and 23 (6.97%) in English. The majority of the studies (325, 98.48%) were authored by researchers from China, while five studies (1.52%) were contributed by authors from other countries, as shown in Figure 2b.

35 SRs on acupuncture for MDD were included, with publication years ranging from 2005 to 2024, as shown in Figure 2a. Of these, 20 were published in Chinese and 15 in English. The first authors were based in various countries, including China, Vietnam, Australia, Taiwan (China), the United States, Norway, and Japan, as illustrated in Figure 2c. The SRs cited between 5 and 64 studies, with an average of 19.09 studies per review. The sample sizes across these SRs ranged from 431 to 7104 participants, with an average of approximately 1576 participants per study.

### 3.3 Population Characteristics in RCTs

Among the RCTs, MDD was the most common primary diagnosis, accounting for 95.76%, followed by treatment‐resistant depression (2.42%) and other types of depression (1.82%). Most studies involved small sample sizes, with 70.30% of studies including 50 to 100 participants, 16.06% including 100 to 300 participants, and 11.52% including fewer than 50 participants. Only 2.12% of studies had a sample size of 300 or more. Regarding the study population's demographics, the age range was not specified in 94.55% of the studies. Research on elderly populations accounted for 3.64%, while studies involving adolescents and children represented 1.52% and 0.30%, respectively. The gender of participants was not reported in 99.09% of the studies, and only 0.96% focused specifically on female participants. In terms of disease severity, 87.58% of studies did not specify the severity of depression. Among those that did, 9.09% focused on mild‐to‐moderate cases, and 0.91% on severe, moderate, mild, and moderate‐to‐severe cases. Regarding TCM syndrome types, 91.82% of studies did not specify a particular syndrome. The most commonly reported syndromes were liver qi stagnation (2.12%), liver qi stagnation with spleen deficiency (1.52%), and yang deficiency (1.21%). Other syndromes such as heart–spleen deficiency, liver qi stagnation with phlegm accumulation, yin deficiency with heat, and phlegm‐qi stagnation were each mentioned in less than 1% of studies. These details are summarized in Table 3.

**TABLE 3:** Population characteristics of the included RCTs (*N* = 330).

Population characteristics	Number	Percentage (%)
**Disease classification**		
Major depressive disorder	316	95.76
Treatment‐resistant depression	8	2.42
Depression with associated symptoms	3	0.91
Masked depression	1	0.30
Primary unipolar depression	1	0.30
Depressive neurosis	1	0.30
**Language**		
Chinese	307	93.03
English	23	6.97
**Sample size**		
≥50 and <100	232	70.30
≥100 and <300	53	16.06
<50	38	11.52
≥300	7	2.12
**Age group**		
Not specified	312	94.55
Elderly	12	3.64
Adolescents	5	1.52
Children	1	0.30
**Gender**		
Not specified	327	99.09
Female	3	0.96
**Severity**		
Not specified	289	87.58
Mild to moderate	30	9.09
Severe	3	0.91
Moderate	3	0.91
Mild	3	0.91
Moderate to severe	2	0.61
**Syndrome differentiation**		
Not specified	303	91.82
Liver qi stagnation	7	2.12
Liver depression and spleen deficiency	5	1.52
Yang deficiency	4	1.21
Heart‐spleen deficiency	3	0.91
Liver qi stagnation and qi obstruction	3	0.91
Yin deficiency with internal heat	1	0.30
Phlegm and qi stagnation	1	0.30
Qi stagnation and blood stasis	1	0.30
Kidney yang deficiency	1	0.30
Qi stagnation transforming into fire	1	0.30

### 3.4 Characteristics of Interventions and Control Measures in RCTs

**FIGURE 3:**
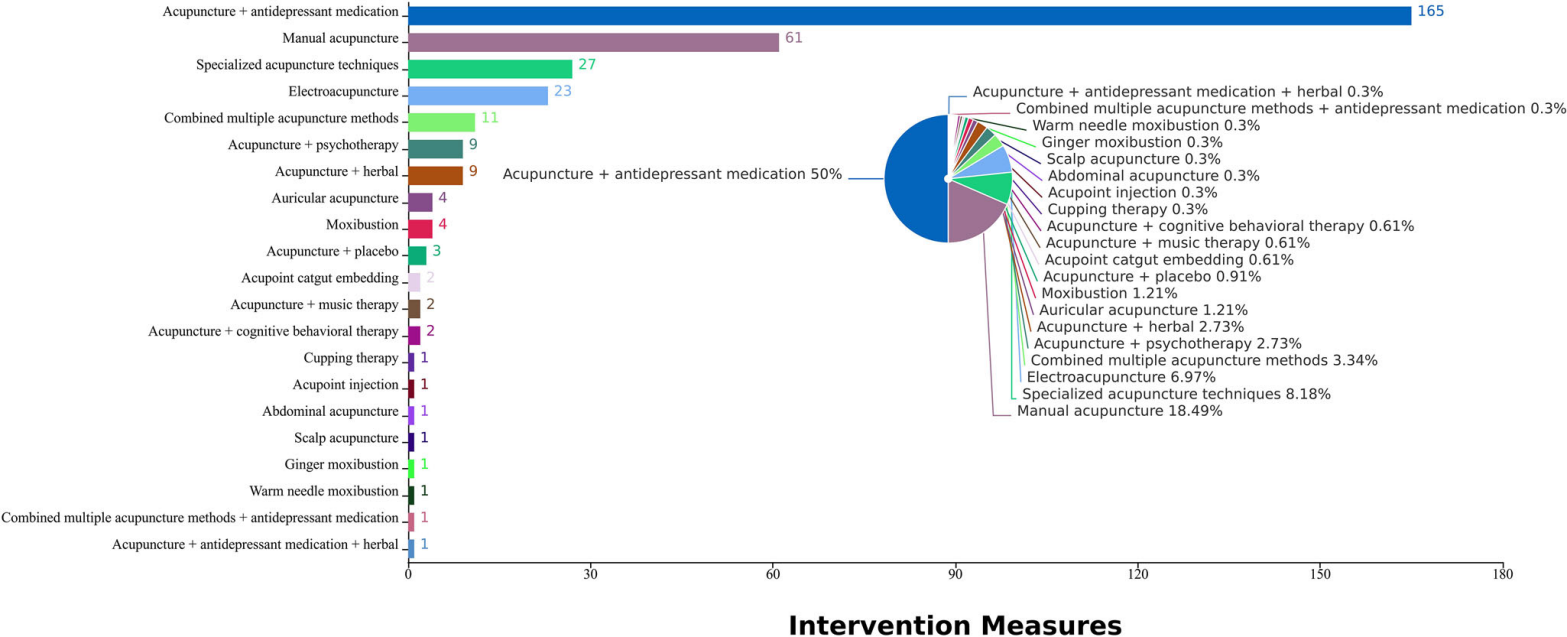
Characteristics of interventions measures in RCTs.

The most commonly used intervention in the included RCTs was combination therapy (203 studies, 61.52%), which included: acupuncture combined with antidepressant medication (165 studies, 50.00%), the use of various acupuncture methods in combination (11 studies, 3.33%), acupuncture combined with herbal (9 studies, 2.73%), psychological therapy (9 studies, 2.73%), cognitive behavioral therapy (2 studies, 0.61%), and music therapy (2 studies, 0.61%). A total of 127 studies (38.48%) employed acupuncture alone as the intervention, with the most common techniques being manual acupuncture (61 studies, 18.48%), special acupuncture techniques (27 studies, 8.18%), and electroacupuncture (23 studies, 6.97%), as shown in Figure 3.

**FIGURE 4:**
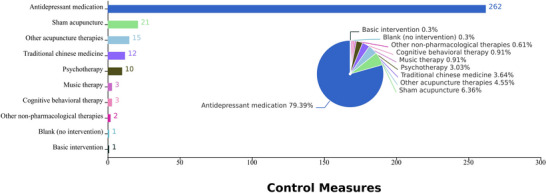
Characteristics of control measures in RCTs.

The most frequently used control measures included antidepressant medication (262 studies, 79.39%), followed by sham acupuncture (21 studies, 6.36%), other acupuncture therapy (15 studies, 4.55%), Chinese herbal medicine (12 studies, 3.64%), and psychological (10 studies, 3.00%). Other control methods such as music therapy, cognitive therapy, non‐pharmacological therapies, blank controls, and basic interventions accounted for less than 1% of studies, as illustrated in Figure 4.

### 3.5 Distribution of Outcome Indicators in RCTs

**FIGURE 5:**
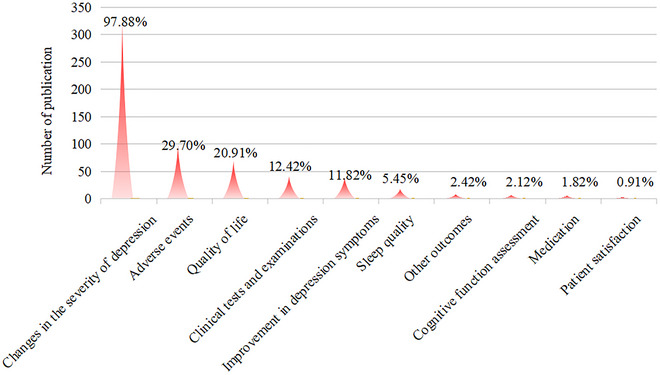
Distribution of outcome indicators in RCTs.

The most commonly evaluated outcome indicator in the RCTs was the change in depression severity, which was reported in 323 studies (97.88%). This was followed by adverse events (98 studies, 29.70%) and quality of life (69 studies, 20.91%). Other outcome indicators included clinical tests and examinations (41 studies, 12.42%), degree of depression improvement (39 studies, 11.82%), and sleep‐related indicators (18 studies, 5.45%). Less frequently reported outcome indicators were cognitive function assessments (7 studies, 2.12%), medication usage (6 studies, 1.82%), and patient satisfaction (3 studies, 0.91%), as shown in Figure 5.

### 3.6 Risk of Bias Assessment in RCTs

**FIGURE 6:**
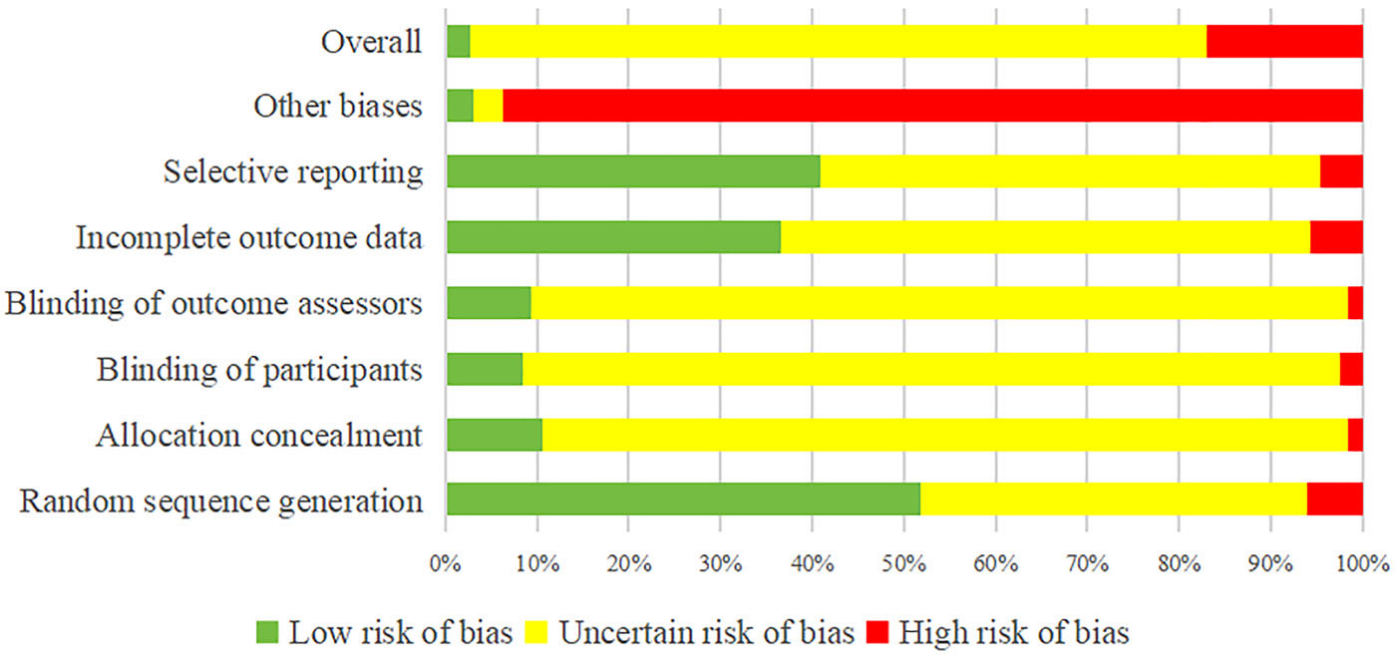
Risk of bias assessment in RCTs based on Cochrane's risk of bias tool.

In the RCTs, 51.82% of studies had a low risk of bias for random sequence generation, 42.12% had an uncertain risk, and 6.06% had a high risk. Most studies lacked sufficient information on allocation concealment, participant blinding, and outcome assessor blinding, with only 10.61%, 8.48%, and 9.39% classified as low risk for these factors, respectively. Nearly 90% had an uncertain risk of bias in these areas. Regarding outcome data completeness, 36.67% of studies had a low risk of bias, 57.58% had an uncertain risk, and 5.76% had a high risk. For selective reporting bias, 40.91% were classified as low risk, while 54.55% had an unclear risk. In other bias categories, 3.03% of studies were low risk, while 93.64% were high risk. Overall, most studies (80.30%) had an unknown risk of bias, followed by those with a high risk (16.97%), and only 2.73% were classified as low risk, as shown in Figure 6.

### 3.7 Clinical Characteristics of SRs

The SRs primarily focused on TCM interventions, such as moxibustion, acupuncture (manual and electroacupuncture), auricular acupressure, and their combination with antidepressant medications (e.g., SSRIs, SNRIs) or other treatments like psychological therapies and music therapy. Overall, the intervention groups consistently demonstrated superior efficacy compared to the control groups (Yan et al. 2021; Leo and Ligot 2007; Armour et al. 2019; Nguyen et al. 2021; Xu et al. 2022; Zhou et al. 2022; Zhang et al. 2016; Wang et al. 2008; Zhang et al. 2016; Chan et al. 2015; Xu et al. 2022; Wang and Chi 2008; Cui 2016; Xu et al. 2022; Meng 2020; Bian et al. 2011; Wang et al. 2020; Lü 2014; Wang et al. 2008; Shen et al. 2014; Yuan et al. 2020; Quang et al. 2017; Xiong et al. 2009; Wang 2021; Fan 2011; Zhong et al. 2008; Peng et al. 2022; Meng et al. 2020; Bai et al. 2024; Li et al. 2024). However, some studies reported no significant differences between the intervention and control groups (Smith et al. 2018; Stub et al. 2011; Mukaino et al. 2005; Sun et al. 2008; Tan et al. 2024).

Regarding outcome indicators, most studies utilized the Hamilton Depression Rating Scale (HAMD) (Leo and Ligot 2007; Xu et al. 2022; Wang et al. 2008; Zhang et al. 2016; Xu et al. 2022; Sun et al. 2008; Meng 2020; Wang et al. 2008; Yuan et al. 2020; Fan 2011; Zhong et al. 2008; Meng et al. 2020; Tan et al. 2024; Bai et al. 2024) or its revised version, the Hamilton Rating Scale for Depression (HRSD) (Stub et al. 2011; Mukaino et al. 2005), as the primary evaluation tool. Other studies assessed indicators such as cure rate (Yan et al. 2021; Wang and Chi 2008; Bian et al. 2011; Peng et al. 2022), remission rate (Smith et al. 2018; Cui 2016), or psychological health status (Quang et al. 2017). The duration of interventions varied widely, ranging from the end of treatment to several weeks or even months post‐treatment.

Most systematic reviews did not provide detailed quality assessments (Yan et al. 2021; Smith et al. 2018; Armour et al. 2019; Stub et al. 2011; Nguyen et al. 2021; Xu et al. 2022; Zhang et al. 2016; Wang et al. 2008; Zhang et al. 2016; Chan et al. 2015; Xu et al. 2022; Wang and Chi 2008; Cui 2016; Sun et al. 2008; Meng 2020; Bian et al. 2011; Wang et al. 2020; Lü 2014; Wang et al. 2008; Shen et al. 2014; Yuan et al. 2020; Quang et al. 2017; Xiong et al. 2009; Wang 2021; Fan 2011; Zhong et al. 2008; Peng et al. 2022; Meng et al. 2020; Tan et al. 2024; Li et al. 2024). A few studies were classified as high quality (Leo and Ligot 2007), while others were rated as moderate (Zhou et al. 2022), low (Mukaino et al. 2005), or even very low quality (Bai et al. 2024). Refer to Table 4 for details.

**TABLE 4:** Clinical characteristics of systematic reviews^a^.

PICO question				First author/year	Number of included RCTs	Intervention group sample size	Control group sample size	Outcome	Effect size (95% confidence interval)/calculation method	Quality of systematic review
Population	Intervention	Control	Outcome indicators/measurement time							
MDD	Moxibustion	Traditional Chinese Medicine, western medicine, music	Cure rate/not reported	Yang JY/2021 (Yan et al. 2021)	24	1154	1100	Intervention outperforms control	2.74[2.11, 3.55]/OR	Not reported
MDD	Acu	Non‐specific acupuncture	HAMD/8 weeks of treatment	Leo RJ/2007 (Leo and Ligot 2007)	4	16	19	Intervention outperforms control	2.44[0.62–9.64]/OR	High quality
MDD	MA	SSRI	Remission status/end of treatment	Smith CA/2018 (Smith et al. 2018)	14	628	704	No significant difference	1.16 [0.98, 1.37]/RR	Not reported
MDD	Acu + SSRI/SNRI	SSRI/SNRI	Depression severity/not reported	Armour M/2019 (Armour et al. 2019)	12	Not reported	Not reported	Intervention outperforms control	0.84(0.61, 1.06)/not reported	Not reported
MDD	EA	Medication	HRSD / Not reported	Stub T/2011 (Stub et al. 2011)	10	352	303	No significant difference	−0.68[−1.49,0.13]/WMD	Not reported
MDD	EA	SSRI	Physical health/end of treatment	Nguyen MD/2021 (Nguyen et al. 2021)	5	150	149	Intervention outperforms control	0.95[0.51,1.38]/MD	Not reported
MDD	Acu + Antidepressant medication	Antidepressants	HAMD‐17 / End of treatment	Xu MM/2022 (Xu et al. 2022)	8	427	376	Intervention outperforms control	−0.98[−1.55, −0.42]/SMD	Not reported
MDD	EA + antidepressant medication	Antidepressants	HAMD‐24/not reported	Zhou Z/2022 (Zhou et al. 2022)	8	Not reported	Not reported	Intervention outperforms control	−6.83[−8.39, −5.27]/WMD	Moderate quality
MDD	AA + other therapies	Other treatments	HRSD/not reported	Zhang ZX/2016 (Zhang et al. 2016)	4	150	147	Intervention outperforms control	−2.95[−4.94, −0.97]/MD	Not reported
MDD	EA + SSRIs	SSRIs	HAMD/1 week after treatment	Wang H/2008 (Wang et al. 2008)	6	215	214	Intervention outperforms control	2.32[1.47, 3.16]/MD	Not reported
Primary depression	EA + SSRIs	SSRIs	HAMD / 1 week after treatment	Zhang Y/2016 (Zhang et al. 2016)	6	215	214	Intervention outperforms control	2.32[1.47,3.16]/MD	Not reported
MDD	EA + SSRIs	SSRIs	Response rate / Not reported	Chan YY/2015 (Chan et al. 2015)	8	263	260	Intervention outperforms control	1.22[1.05, 1.42]/RR	Not reported
MDD	Acu	Medication	HAMD / 8 acupuncture sessions	Guixing X/2022 (Xu et al. 2022)	57	Not reported	Not reported	Intervention outperforms control	17.68 (14.23–21.13)/not reported	Not reported
MDD	EA	Antidepressants	HRSD / Not reported	Mukaino Y/2005 (Mukaino et al. 2005)	4	Not reported	Not reported	No significant difference	−0.43(−5.61, −4.76)/WMD	Low quality
MDD	EA	Amitriptyline	Cure rate / Not reported	Wang L/2008 (Wang and Chi 2008)	4	289	321	Intervention outperforms control	−0.92[−1.46, −0.39]/WMD	Not reported
MDD	AA + Other therapies	Single other therapy	Remission status / Not reported	Cui HJ/2016 (Cui 2016)	4	138	138	Intervention outperforms control	3.87[2.10, 7.13]/OR	Not reported
MDD	MA	Fluoxetine	HAMD / 6 weeks	Sun YL/2008 (Sun et al. 2008)	4	174	139	No significant difference	−0.19[−1.51, 1.13]/WMD	Not reported
MDD	MA + antidepressants	Antidepressant	HAMD factor I/not reported	Xu S/2022 (Xu et al. 2022)	4	267	187	Intervention outperforms control	−0.80[−1.21, −0.39]/MD	Low quality or high quality
MDD	Acu + Medication	Medication group	HAMD‐17/4 weeks of treatment	Meng Y/2020 (Meng 2020)	22	826	795	Intervention outperforms control	4.06[3.36, 4.76]/MD	Not reported
MDD	MA	Medication	Cure rate / 4 weeks of treatment	Bian XK/2011 (Bian et al. 2011)	14	575	502	Intervention outperforms control	2.65[2.04, 3.43]/OR	Not reported
MDD	MA	Medication	Significant cure rate/not reported	Wang YC/2020 (Wang et al. 2020)	16	754	596	Intervention outperforms control	1.79[1.40, 2.28]/OR	Not reported
MDD	MA	Medication	HAMD different scores/not reported	Lü Z/2014 (Lü 2014)	15	727	569	Intervention outperforms control	1.27[−1.47, −1.08]/MD	Not reported
MDD	MA	Amitriptyline	HAMD/after treatment	Wang L/2008 (Wang et al. 2008)	3	255	287	Intervention outperforms control	−0.93[−1.48, −0.38]/WMD	Not reported
MDD	Acu	Antidepressant	Effectiveness/not reported	Shen H/2014 (Shen et al. 2014)	13	450	434	Intervention outperforms control	1.69[1.13, 2.52]/OR	Not reported
MDD	Acu + SSRIs	SSRIs	HAMD‐17/2 weeks of treatment	Yuan T/2020 (Yuan et al. 2020)	3	448	426	Intervention outperforms control	−2.43[3.43, −1.43]/MD	Not reported
MDD	EA	Medication	Psychological domain/6 weeks of treatment	Nguyen BQ/2017 (Quang et al. 2017)	5	150	149	Intervention outperforms control	1.45[1.07, 1.85]/MD	Not reported
MDD	Acu	Fluoxetine	Effectiveness/12 weeks of treatment	Xiong J/2009 (Xiong et al. 2009)	5	337	338	Intervention outperforms control	1.15[1.07, 1.22]/RR	Not reported
MDD	MA + Western medicine	Western Medicine	Effectiveness/not reported	Wang CB/2021 (Wang 2021)	24	Not reported	Not reported	Intervention outperforms control	3.69,[2.68, 5.09]/OR	Not reported
MDD	Comprehensive acupuncture therapy (MA, AA, EA, SA, massage, abdominal acupuncture)	Antidepressants, acupuncture, auricular acupuncture, EA, sham acupuncture	HAMD/not reported	Fan L/2010 (Fan 2011)	16	737	720	Intervention outperforms control	−1.92[−3.27, −0.53] /WMD	Not reported
MDD	MA + AA	Adjacent sham acupoints + auricular acupuncture	HAMD/not reported	Zhong BL/2008 (Zhong et al. 2008)	3	234	143	Intervention outperforms control	−4.79[−6.17, −3.14]/WMD	Not reported
MDD	MA +Medication	Medication	Cure rate/not reported	Peng HY/2022 (Peng et al. 2022)	13	504	502	Intervention outperforms control	1.12(1.07, 1.17)/RR	Not reported
MDD	MA + Medication	Medication	HAMD/4 weeks of treatment	Meng Y/2020 (Meng et al. 2020)	21	825	773	Intervention outperforms control	2.93[2.23, 3.62]/WMD	Not reported
MDD	MA	Medication	HAMD/4 weeks of treatment	Tan Y/2024 (Tan et al. 2024)	9	294	294	No significant difference	−2.15[−4.23, −0.07]/RR	Not reported
MDD	Acu + Medication	Medication	HAMD/after treatment	Bai JY/2024 (Bai et al. 2024)	5	267	267	Intervention outperforms control	3.08[−4.46, −1.7]/MD	Extremely low quality
MDD	MA	Medication	HAMD effectiveness/after treatment	Li WT/2024 (Li et al. 2024)	15	472	434	Intervention outperforms control	1.09[1.03, 1.153] /RR	Not reported

Abbreviations: AA: auricular acupuncture; Acu: acupuncture; EA: electroacupuncture; MA: manual acupuncture; MD: mean difference; OR: odds ratio; RR: relative risk; SMD: standardized mean difference; WMD: weighted mean difference.

^a^ For studies with multiple meta‐analyses, only one meta‐analysis is selected for display.

### 3.8 Evidence Distribution and Methodological Quality of Included Studies in SRs

**FIGURE 7:**
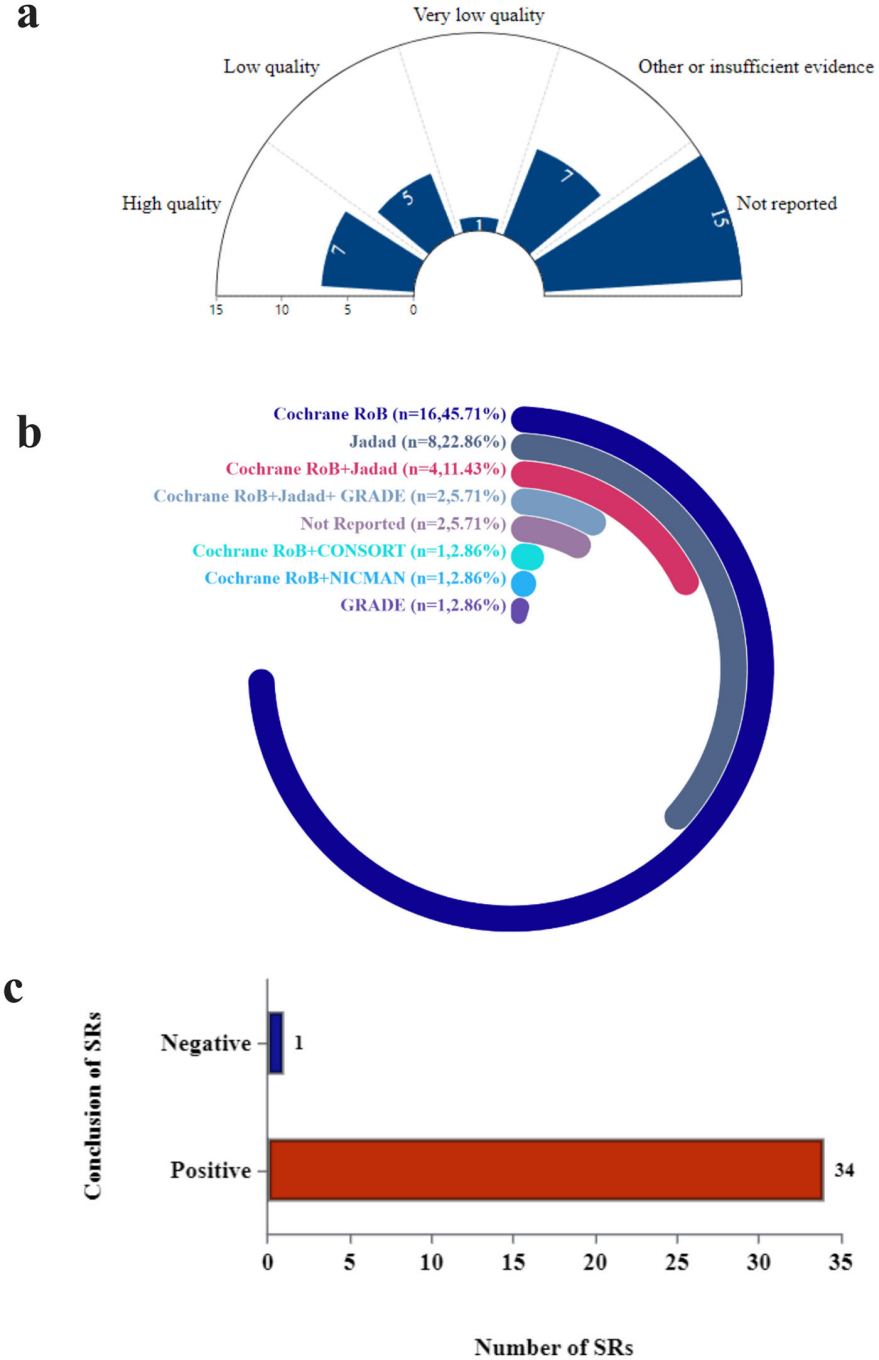
Evidence distribution and methodological quality of included studies in systematic reviews. (a) Quality of included studies in SRs. (b) Quality assessment tools used in SRs. (c) Conclusions of SRs.

Among the 35 SRs included, 7 (20%) clearly reported that the RCTs incorporated were of high quality, 5 (14.29%) included RCTs of low quality, and 1 (2.86%) included RCTs of very low quality. Fifteen SRs (42.86%) did not report the quality of the included RCTs. Additionally, there were instances where the quality categorization of the RCTs was ambiguous. Overall, the distribution of evidence quality was quite varied, with a significant proportion of SRs not explicitly reporting the quality of the included trials (see Figure 7a).

In terms of quality assessment tools, the Cochrane RoB tool was the most commonly used, applied in 16 reviews (45.71%). The Jadad scale followed as the second most frequent tool, used in eight reviews (22.86%). Some SRs employed a combination of the Cochrane RoB and Jadad scales (four reviews, 11.43%), while two reviews (5.71%) used a combination of Cochrane RoB, Jadad, and GRADE. Additionally, two reviews (5.71%) did not report the quality assessment tools used. A very small number of SRs employed other tool combinations, such as Cochrane RoB with CONSORT, Cochrane RoB with NICMAN, and Cochrane RoB with GRADE, with each combination being used in just one review (2.86%), as shown in Figure 7b.

Finally, 34 SRs (97.14%) concluded that acupuncture has potential benefits for the treatment of MDD, supporting its positive therapeutic effects, as shown in Figure 7c.

### 3.9 Overview of SRs

Based on AMSTAR‐2, the quality reassessment of the systematic reviews shows that the overall quality of 35 SRs was relatively low. Among these, 26 studies (74.29%) were classified as very low quality (Yan et al. 2021; Leo and Ligot 2007; Nguyen et al. 2021; Zhang et al. 2016; Wang et al. 2008; Zhang et al. 2016; Xu et al. 2022; Mukaino et al. 2005; Wang and Chi 2008; Cui 2016; Sun et al. 2008; Xu et al. 2022; Bian et al. 2011; Shen et al. 2014; Yuan et al. 2020; Quang et al. 2017; Xiong et al. 2009; Wang 2021; Fan 2011; Zhong et al. 2008; Peng et al. 2022; Meng et al. 2020; Tan et al. 2024; Bai et al. 2024; Li et al. 2024), while 8 studies (22.86%) were categorized as low quality (Armour et al. 2019; Stub et al. 2011; Xu et al. 2022; Zhou et al. 2022; Chan et al. 2015; Meng 2020; Wang et al. 2020; Lü 2014), and only 1 study (2.86%) was considered high quality (Smith et al. 2018).

Most studies performed well on defining research questions and methodological design. For instance, item 1 (Inclusion criteria, such as PICO) and 3 (Types of studies) both received full marks (100%), indicating clear adherence to standard procedures. However, item 2 (Protocol development and adherence) showed that only four studies (11.43%) provided a protocol and addressed deviations, while the majority (65.71%) only partially met the criteria.

In literature search and data quality control, item 4 (Comprehensive search strategy) demonstrated that only six studies (17.14%) used a thorough search strategy, including gray literature and references, with 80% partially meeting the requirements. Item 5 (Dual independent screening) and item 6 (Dual independent data extraction) showed better adherence, with 77.14% meeting the standards for data extraction, but lower compliance (31.43%) for literature screening. Item 7 (Exclusion list and reasons) had nearly all studies failing to provide exclusion lists, severely limiting transparency.

On bias assessment, item 9 (Bias risk tool) showed that 85.71% of studies used appropriate tools, but item 12 (Impact of bias on results) and item 13 (Discussion of bias) revealed that 25.71% of studies did not adequately assess or discuss bias, undermining confidence in their findings. Regarding heterogeneity and publication bias, item 14 (Heterogeneity analysis) showed 65.71% of studies provided reasonable explanations, while item 15 (Publication bias) indicated that just over half (51.43%) addressed this issue. Finally, in terms of transparency, item 10 (Funding sources) showed no studies disclosed funding sources, and item 16 (Conflict of interest) revealed that only 28.57% clearly reported potential conflicts, affecting research independence.

**FIGURE 8:**
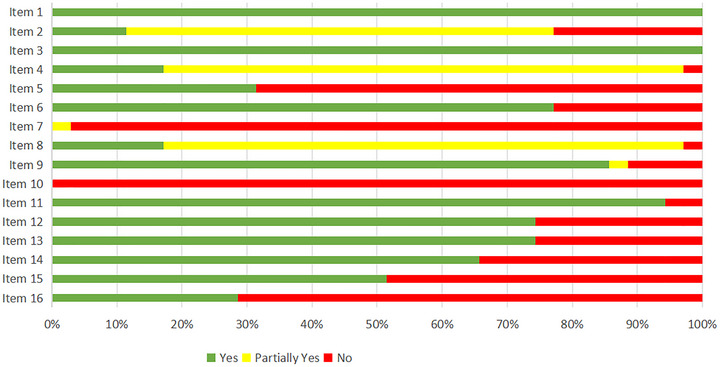
Quality assessment of systematic reviews based on AMSTAR‐2.

In summary, while SRs excelled in areas like research clarity and bias assessment (e.g., items 1, 3, 9, 12, and 13), there were significant gaps in protocol development, search comprehensiveness, exclusion transparency, and conflict of interest reporting, as shown in Figure 8.

### 3.10 Evidence Distribution in Clinical Guidelines

The 2014 *Evidence‐Based Guidelines of Clinical Practice With Acupuncture and Moxibustion: Depression (revised)* from the Chinese Acupuncture Association recommended the “calming the mind and soothing the liver” approach, particularly for both general and special populations (e.g., post‐stroke, postpartum, and menopausal depression). Treatment options recommended include electroacupuncture, manual acupuncture, auricular acupuncture, among others, with selection based on individual patient factors, such as tolerance to electroacupuncture, somatization symptoms, and sleep disorders (China Association of Acupuncture‐Moxibustion 2014).

In 2023, the World Federation of Acupuncture and Moxibustion Societies (WFAS) issued the *Clinical Guideline on Acupuncture and Moxibustion: Adult Major Depressive Disorder (Mild–Moderate Degree*) providing several conditional recommendations, though with low‐quality evidence. The WFAS expert panel recommended psychotherapy as the first‐line treatment and suggested combining acupuncture with antidepressants if chosen. Recommended acupuncture methods include electroacupuncture and manual, auricular, or body acupuncture, with treatment sessions scheduled at least three times a week for up to 4 weeks (Zhao et al. 2023).

Recommendations for acupuncture in the treatment of depression are not consistent across comprehensive clinical guidelines. The American College of Physicians (ACP) 2016 guidelines did not endorse acupuncture as a standalone treatment and offered no clear guidance on comparing acupuncture with antidepressant medications, citing low‐quality evidence (Qaseem et al. 2016).

Conversely, the Canadian Network for Mood and Anxiety Treatments (CANMAT) 2016 guidelines positioned acupuncture as a third‐line adjunctive treatment for mild‐to‐moderate MDD (based on Level 2 evidence) (Ravindran et al. 2016). Similarly, the American Psychological Association (APA) 2019 guidelines recommended acupuncture as a supplementary treatment alongside antidepressant medications but conclude that the evidence was insufficient to support acupuncture as a monotherapy (Guideline Development Panel for the Treatment of Depressive Disorders 2019).

In 2022, the US Department of Veterans Affairs and Department of Defense (VA/DoD) guidelines explicitly advised against the use of acupuncture for depression due to inadequate evidence (U.S. Department of Veterans Affairs, U.S. Department of Defense 2022). The Ministry of Health Malaysia echoed this stance, highlighting the lack of high‐quality controlled trials, particularly those involving sham acupuncture or placebo controls, and consequently not recommending acupuncture for depression treatment (Ministry of Health Malaysia 2019).

In contrast, the 2023 *Guidelines for the Integrated Traditional Chinese and Western Medicine Diagnosis and Treatment of Depression* in China adopted a more favorable view of acupuncture. These guidelines particularly support the use of acupuncture for patients experiencing comorbid anxiety, sleep disturbances, and somatic symptoms alongside depression. Additionally, the guidelines noted that combining acupuncture with herbal can enhance therapeutic outcomes, demonstrating a comprehensive approach to depression treatment (China Association of Integrative Medicine, Chinese Medical Association 2023). These details are summarized in Table 5.

**TABLE 5:** Evidence distribution in clinical guidelines.

Guideline title	Country	Year	Population	Recommendation/not recommended	Evidence and evidence level	Grading criteria
**Guidelines recommending acupuncture**						
CANMAT 2016 Clinical Guidelines for the Management of Adults with Major Depressive Disorder: Section 5. Complementary and Alternative Medicine Treatments (Ravindran et al. 2016)	Canada	2016	Adult major depressive disorder	Acupuncture is recommended as a third‐line treatment in adjunctive therapy for mild to moderate MDD.	Two well‐designed RCTs; four systematic reviews (Level 2)	CANMAT‐defined criteria
Clinical Practice Guideline for the Treatment of Depression Across Three Age Cohorts. American Psychological Association Guideline Development Panel for the Treatment of Depressive Disorders (Guideline Development Panel for the Treatment of Depressive Disorders 2019)	USA	2019	Depressive disorders (including major depression, subsyndromal depression, and persistent depressive disorder). General adult population of depression	If considering adjunctive treatments, acupuncture is suggested to be added to antidepressants; insufficient evidence for acupuncture monotherapy.	Four well‐designed RCTs; four systematic reviews (Level 2)	GRADE
Integrated Chinese and Western Medicine Guidelines for the Treatment of Depression	China	2023	Depression	(1) Co‐occurring anxiety: acupuncture (4 RCTs, evidence level: D, strong recommendation); (2) co‐occurring sleep disorders: acupuncture (69 RCTs, evidence level: C, strong recommendation); (3) co‐occurring somatic symptoms: acupuncture (3 RCTs, evidence level: C, weak recommendation); (4) perinatal women: acupuncture (1 RCT, evidence level: C, weak recommendation); (5) perimenopausal women: acupuncture (17 RCTs, evidence level: C, strong recommendation); (6) elderly population: acupuncture (1 RCT, evidence level: C, strong recommendation); (7) acute phase of	Not reported	GRADE
				depression with liver qi stagnation: acupuncture (8 RCTs, evidence level: C, strong recommendation); (8) acute phase of depression with liver qi stagnation and spleen deficiency: acupuncture combined with Western medicine (7 RCTs, evidence level: C, strong recommendation); (9) depression consolidation and maintenance phase: acupuncture (1 RCT, evidence level: C, strong recommendation).		
**Guidelines not recommending acupuncture/insufficient evidence**						
Nonpharmacologic Versus Pharmacologic Treatment of Adult Patients with Major Depressive Disorder: A Clinical Practice Guideline from the American College of Physicians (Qaseem et al. 2016)	USA	2016	Adult major depressive disorder	No recommendation for comparison of second‐generation antidepressants vs. acupuncture; no recommendation for comparison of combined second‐generation antidepressants and acupuncture vs. antidepressants alone.	Acupuncture monotherapy: two well‐designed RCTs (low); acupuncture combined: one well‐designed RCT (low)	GRADE
Practice Guideline for the Treatment of Patients with Major Depressive Disorder (3rd edition) (The Work Group on Major Depressive Disorder 2010)	USA	2010	Major depressive disorder	No recommendation due to insufficient evidence.	Two RCTs; one systematic review	Roman numeral
VA/DoD Clinical Practice Guideline for the Management of Major Depressive Disorder (U.S. Department of Veterans Affairs, U.S. Department of Defense 2022)	USA	2022	Major depressive disorder	Not recommended due to insufficient evidence.	Two systematic reviews	GRADE
Management of Major Depressive Disorder (2nd edition) (Ministry of Health Malaysia 2019)	Malaysia	2019	Major depressive disorder, Post‐stroke depression	Not recommended due to insufficient evidence: lack of sham acupuncture/placebo/waiting list/control treatments.	One systematic review with 30 RCTs; one subgroup analysis	GRADE

## 4 Discussion

Our overview comprehensively synthesized evidence from 330 RCTs, 35 SRs, and 9 clinical guidelines on acupuncture for MDD. Overall, acupuncture demonstrated potential benefits in reducing depressive symptom severity, particularly when used as an adjunctive therapy to antidepressants. Some evidence also supports additional benefits for subgroups with comorbid anxiety, sleep disturbances, and somatic symptoms.

In RCTs, acupuncture was most frequently studied in combination with antidepressants, where it often showed greater improvements in symptom severity compared to medication alone. Manual acupuncture was the most commonly studied technique, followed by special acupuncture and electroacupuncture. While outcome measures predominantly focused on symptom severity (97.88% of RCTs), some trials also reported improvements in comorbid anxiety, sleep disturbances, and somatic symptoms, supporting the potential utility of acupuncture for specific subgroups of patients. Quality of life was evaluated in 20.91% of RCTs, with findings suggesting potential improvements, although data remain limited. Long‐term outcomes, recurrence rates, and patient‐centered measures such as satisfaction or treatment adherence were rarely assessed, representing an important evidence gap. Safety outcomes were less consistently reported, but available data indicated that acupuncture was generally well tolerated, with no significant differences in serious adverse events compared to controls. Despite the growing volume of studies, methodological weaknesses remain a critical challenge. Risk of bias assessments revealed that although 51.82% of RCTs had a low risk in random sequence generation, nearly 90% failed to adequately report allocation concealment, blinding, or outcome assessor blinding, undermining confidence in the findings. Only 36.67% of trials reported complete outcome data, while selective reporting bias was unaddressed in more than half. These findings highlight the importance of stricter adherence to CONSORT reporting guidelines in future trials. Additional limitations stem from the characteristics of the included studies. The majority of RCTs were conducted in China, typically with small sample sizes (50–100 participants) and single‐center designs. Most trials did not stratify by age, disease severity, or TCM syndrome type. Although women are twice as likely as men to develop MDD (Kuehner 2017), only 0.96% of studies specifically examined female populations.

Among the 35 SRs included, most reported favorable effects of acupuncture, particularly in combination with antidepressants. However, the quality varied substantially. Reassessment with AMSTAR‐2 revealed that 74.29% of SRs were rated very low in methodological quality. A major limitation was the inadequate assessment of bias: 68.57% of SRs failed to fully evaluate how risk of bias in the primary studies might influence conclusions. Additionally, only 11.43% of SRs addressed publication bias, suggesting that unpublished studies may skew the overall findings. Transparency was also lacking, with many reviews failing to report excluded studies or funding sources, raising concerns regarding independence. Overall, while SRs generally adhere to basic methodological standards (e.g., the PICO framework and study type specifications), improvements are needed in transparency, bias control, and reporting quality. These deficiencies undermine reproducibility and comparability. Future SRs should adopt standardized protocols, ensure transparent reporting, and systematically address bias and heterogeneity to provide more reliable evidence for clinical decision‐making.

Clinical practice guidelines showed marked variability in recommendations. Chinese guidelines, such as those from the Chinese Acupuncture Society and integrated TCM‐Western medicine guidelines, generally endorsed acupuncture as an effective adjunctive therapy, particularly for subgroups with comorbid anxiety, sleep disturbances, or somatic symptoms. In contrast, international guidelines (APA, CANMAT) tended to recommend acupuncture as a third‐line adjunct at best, while others (ACP, VA/DoD, Malaysia) did not recommend it due to insufficient evidence. These discrepancies reflect both differences in grading systems (e.g., GRADE, CANMAT‐defined criteria, and Roman numerals) and the generally low certainty of evidence cited. Importantly, guidelines that recommend acupuncture consistently position it as an adjunctive, third‐line strategy, while non‐recommending guidelines mostly cite insufficient high‐quality evidence rather than proven ineffectiveness. This underscores the central challenge: the lack of robust, large‐scale RCTs rather than conflicting interpretations of strong evidence. For clinicians, this suggests that acupuncture may be considered cautiously as a complementary option, with greater weight given to guidelines that employ transparent grading frameworks and recent evidence assessments, alongside consideration of local practice settings and patient preferences.

## 5 Strengths and Limitations

This study has several strengths. It is, to our knowledge, the most comprehensive synthesis of RCTs, SRs, and guidelines on acupuncture for MDD to date. Screening, data extraction, and quality assessment were performed independently by two reviewers, enhancing reliability, and the use of multiple quality appraisal tools (Cochrane RoB, AMSTAR‐2) allowed cross‐validation of methodological rigor across study types.

Several limitations should also be acknowledged. We restricted our search to Chinese and English publications, which cover the majority of acupuncture‐related literature and could be reliably assessed by our team, but this may have introduced language bias. Moreover, most included trials were conducted in China, raising concerns about regional bias and limited external validity. Cultural practices, patient expectations, and health system differences (e.g., TCM integration and reimbursement policies) may influence trial design and outcomes, limiting generalizability to non‐Chinese contexts. To address this gap, future research should prioritize large‐scale, multicenter, and multinational RCTs with harmonized protocols to validate the effectiveness of acupuncture for MDD in more diverse populations and health care contexts. We also relied on published data without contacting study authors, which may have left missing information unresolved. Gray literature was excluded to ensure methodological rigor, but this may have increased reporting bias. Finally, our use of the original Cochrane RoB tool, rather than the newer RoB 2, was necessary for consistency across older trials, though future reviews may benefit from applying more advanced tools when reporting quality allows.

## 6 Conclusion

Evidence to date suggests that acupuncture may offer therapeutic benefits for MDD, particularly as an adjunctive treatment, and may be most beneficial for patients with comorbid symptoms such as anxiety, sleep disturbances, and somatic complaints. However, the overall body of evidence is weakened by small sample sizes, methodological weaknesses, narrow outcome measures, and the regional concentration of trials. Systematic reviews often suffer from poor transparency and inadequate bias assessment, while guideline recommendations remain inconsistent due to the limited certainty of evidence. Future research should prioritize large, multicenter, multinational RCTs with rigorous design, standardized protocols, and long‐term follow‐up. Standardization of outcome measures, incorporation of patient‐centered endpoints, and adherence to international reporting standards will be essential to improving evidence quality. Only through such efforts can acupuncture's role in MDD treatment be more definitively established and integrated into clinical guidelines worldwide.

## Author Contributions

**Han Tang**: data curation, writing – original draft, writing – review and editing, formal analysis. Yi **Gou**: data curation, writing – review and editing, formal analysis. Xiao‐yi **Hu**: methodology, investigation, writing – review and editing. Zhen **Luo**: methodology, investigation, writing – review and editing. Wei‐juan **Gang**: conceptualization, supervision, writing – review and editing, project administration. Hong **Zhao**: conceptualization, supervision, writing – review and editing, resources.

## Funding

The study was supported by the National Natural Science Foundation of China (No. 82474644), the Sanming Project of Medicine in Shenzhen (No. SZZYSM202101007), the Science and Technology Innovation Engineering Program of the China Academy of Chinese Medical Sciences (No. CI2021A03503); the National Key Research and Development Program of China (No. 2017YFC1703606); and the Luohu Soft Science Research Program (No. LX202202128). The funding source had no role in the study design, data collection, data analysis, data interpretation, writing of the report, or submission of the report as an article for publication.

## Conflicts of Interest

The authors declare no conflicts of interest.

## Supporting information




**Supplementary Materials**: brb371075‐sup‐0001‐AppendixA.pdf.

## Data Availability

The data that support the findings of this study are available from the corresponding author upon reasonable request.
