# Supplementary material for: Acupuncture for Patients With Major Depressive Disorder: An Evidence Map of Randomized Controlled Trials, Systematic Reviews, and Clinical Guidelines
Source: Brain Behav. 2025 Nov 30;15(12):e71075. doi: 10.1002/brb3.71075 (PMC12665193; doi:10.1002/brb3.71075)
Supplement: Supplementary file 1 — Supplementary Materials: brb371075‐sup‐0001‐AppendixA.pdf. [file BRB3-15-e71075-s001.pdf]

Appendix A. Search strategy

Search strategy of Pubmed

1 Acupuncture or Acupuncture Therapy or Auriculotherapy or Moxibustion or dry needling[MeSH Terms]

2 acupunct*[Text Word] OR acupoint*[Text Word] OR electroacupunct*[Text Word] OR electro - acupunct*[Text Word] OR auriculotherap*[Text Word] OR auriculoacupunct*[Text Word] OR moxibust*[Text Word] OR scalpacupunct*[Text Word]

3 #1 OR #2

4 depress*[Title]

5 Depression OR Depression Disorder OR Major Depressive Disorder, Major[MeSH Major Topic]

6 #4 OR #5

7 Randomized Controlled Trial[Publication Type]

8 controlled clinical trial[Title/Abstract] OR RCT[Title/Abstract] OR random*[Title/Abstract] OR allocat*[Title/Abstract] OR assign*[Title/Abstract] OR placebo[Title/Abstract]

9 #7 OR #8

10 animal[MeSH Terms]

11 animal[Title/Abstract] OR rat[Title/Abstract] OR mice[Title/Abstract] OR dog[Title/Abstract] OR pig[Title/Abstract] OR rabbit[Title/Abstract] OR fish[Title/Abstract]

12 #10 OR #11

13 #3 AND #6 AND #9

14 #13 NOT #12

Search strategy of Embase

#1 ‘acupuncture’ OR ‘electroacupuncture’ OR ‘electrical stimulation’ OR ‘body acupuncture’ OR ‘auricular acupuncture’ OR ‘auricular needle’ OR ‘ear acupuncture’ OR ‘auricular plaster therapy’ OR ‘auricular point sticking’ OR ‘scalp acupuncture’ OR ‘scalp sensory’ OR ‘scalp stimulation’ OR ‘dry needle’ OR ‘warm acupuncture moxibustion’ OR ‘moxibustion’ OR ‘acupuncture injection’

#2 ('depression' OR 'depression disorder' OR 'major depressive disorder'/exp)

#3'randomized controlled trial' OR 'controlled clinical trial' OR 'rct' OR 'random*' OR 'allocat*' OR 'assign*' OR 'placebo' AND [<1966-2024]/py

#4 #1 AND #2 AND #3 AND [humans]/lim AND [<1966-2024]/py AND ([systematic review]/lim OR [meta analysis]/lim OR [randomized controlled trial]/lim OR 'controlled clinical trial'/de)

Search strategy of Cochrane & CENTRAL

#1 Acupuncture

#2 Acupuncture Therapy

#3 Auriculotherapy

#4 Moxibustion

#5 dry needling

#6 #1 OR #2 OR #3 OR #4 OR #5

#7 (acupunct*):ti,ab,kw OR (acupoint*):ti,ab,kw OR (electroacupunct*):ti,ab,kw OR (electro-acupunct*):ti,ab,kw OR (auriculotherap*):ti,ab,kw OR (auriculoacupunct*):ti,ab,kw OR (moxibust*):ti,ab,kw OR (scalpacupunct*):ti,ab,kw

#8 #6 OR #7

#9 depression

#10 (depress*):ti,ab,kw OR (depression disorder):ti,ab,kw OR (major depressive disorder):ti,ab,kw

#11#9 OR #10

#12 Randomized Controlled Trial

#13 (controlled clinical trial):ti,ab,kw OR (RCT):ti,ab,kw OR (random*):ti,ab,kw OR (allocat*):ti,ab,kw OR (assign*):ti,ab,kw OR (placebo):ti,ab,kw

#14 #12 OR #13

#15 #8 AND #11 # AND #14

Search strategy of CNKI, WANFANG Data, VIP, and Sinomed are available from the corresponding author.
